# Supplementary material for: The Fibromyalgia Decomposition Phenomenon: A Reflexive Thematic Analysis
Source: Behav Sci (Basel). 2024 Jan 11;14(1):47. doi: 10.3390/bs14010047 (PMC10813499; doi:10.3390/bs14010047)
Supplement: Supplementary file 1 [file behavsci-14-00047-s001.zip › behavsci-2756234-supplementary.pdf]

Data analysis - the six-phase analytical process

Phase one: familiarisation with the data

Reviewer 1 and Reviewer 3 undertook interview transcription from 6 February 2022 through to 29 July 2022. Since the transcribers had undertaken the semi-structured audio-recorded interviews it meant that immersion in the dataset could occur from an early stage. Reviewer 1 could visualise the interviewee and their mannerisms, pauses, laughs, and cries whilst transcribing, which really allowed the data to come alive. Reviewer 1 began making comments on the data at this point. Reviewer 2 then made comments blind to Reviewer 1 for Participants A-E, for both their First Interview and their Treatment Visit 9 interview (total of 10 interviews at this point) (Figure S1).

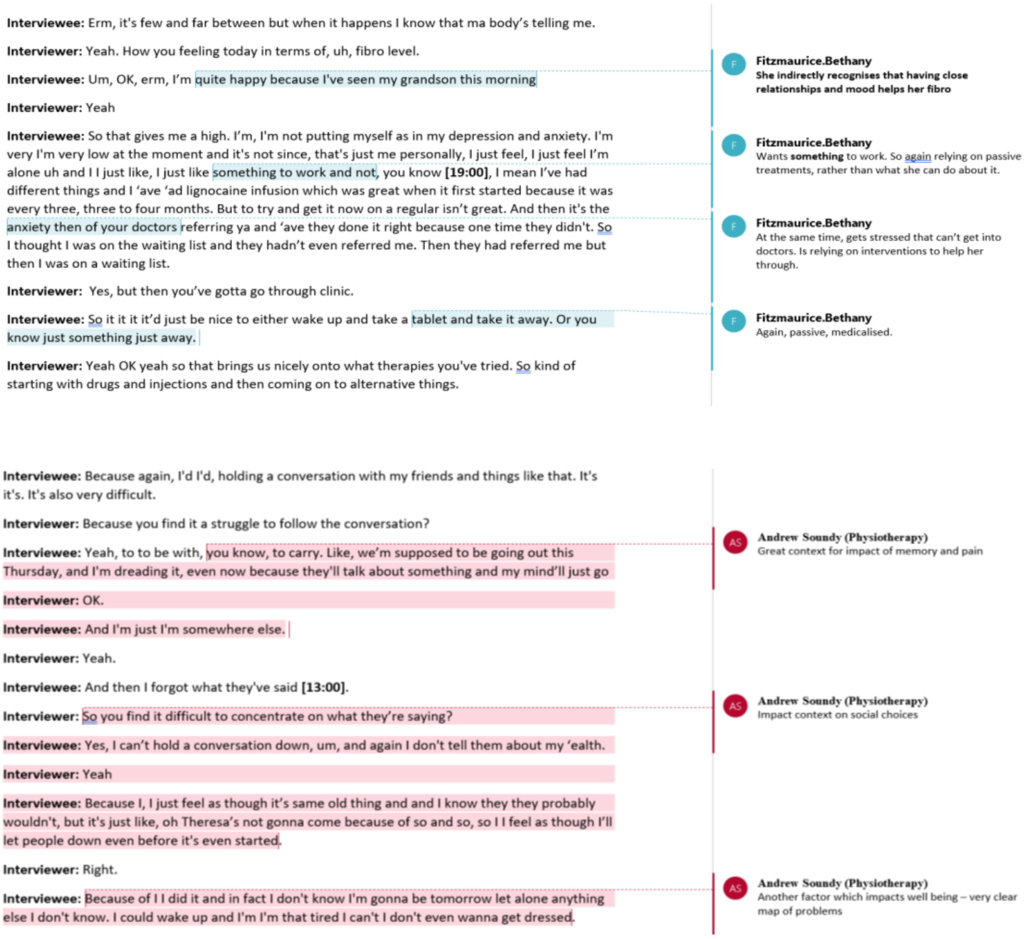
Interviewee: Erm, it's few and far between but when it happens I know that ma body's telling me.

Interviewer: Yeah. How you feeling today in terms of, uh, fibro level.

Interviewee: Um, OK, erm, I'm quite happy because I've seen my grandson this morning

Interviewer: Yeah

Interviewee: So that gives me a high. I'm, I'm not putting myself as in my depression and anxiety. I'm very I'm very low at the moment and it's not since, that's just me personally, I just feel, I just feel I'm alone uh and I I just like, I just like something to work and not, you know **[19:00]**, I mean I've had different things and I 'ave 'ad lignocaine infusion which was great when it first started because it was every three, three to four months. But to try and get it now on a regular isn't great. And then it's the anxiety then of your doctors referring ya and 'ave they done it right because one time they didn't. So I thought I was on the waiting list and they hadn't even referred me. Then they had referred me but then I was on a waiting list.

Interviewer: Yes, but then you've gotta go through clinic.

Interviewee: So it it it it'd just be nice to either wake up and take a tablet and take it away. Or you know just something just away.

Interviewer: Yeah OK yeah so that brings us nicely onto what therapies you've tried. So kind of starting with drugs and injections and then coming on to alternative things.

Fitzmaurice.Bethany
She indirectly recognises that having close relationships and mood helps her fibro

Fitzmaurice.Bethany
Wants **something** to work. So again relying on passive treatments, rather than what she can do about it.

Fitzmaurice.Bethany
At the same time, gets stressed that can't get into doctors. Is relying on interventions to help her through.

Fitzmaurice.Bethany
Again, passive, medicalised.

Interviewee: Because again, I'd I'd, holding a conversation with my friends and things like that. It's it's. It's also very difficult.

Interviewer: Because you find it a struggle to follow the conversation?

Interviewee: Yeah, to to be with, you know, to carry. Like, we'm supposed to be going out this Thursday, and I'm dreading it, even now because they'll talk about something and my mind'll just go

Interviewer: OK.

Interviewee: And I'm just I'm somewhere else.

Interviewer: Yeah.

Interviewee: And then I forgot what they've said **[13:00]**.

Interviewer: So you find it difficult to concentrate on what they're saying?

Interviewee: Yes, I can't hold a conversation down, um, and again I don't tell them about my 'ealth.

Interviewer: Yeah

Interviewee: Because I, I just feel as though it's same old thing and and I know they they probably wouldn't, but it's just like, oh Theresa's not gonna come because of so and so, so I I feel as though I'll let people down even before it's even started.

Interviewer: Right.

Interviewee: Because of I I did it and in fact I don't know I'm gonna be tomorrow let alone anything else I don't know. I could wake up and I'm I'm that tired I can't I don't even wanna get dressed.

Andrew Soundy (Physiotherapy)
Great context for impact of memory and pain

Andrew Soundy (Physiotherapy)
Impact context on social choices

Andrew Soundy (Physiotherapy)
Another factor which impacts well being – very clear map of problems

Figure S1. Snapshot of Reviewer 1 and Reviewer 2 initial 'blind' comments on interview transcripts.

Phase two: generating initial codes

Reviewer 1 and 2 underwent the first in a series of regular meetings. At this early stage in the process, we formed both an 'Agreement of Procedures' and an 'Agreed theme generation process'. Here, it was decided that Reviewer 1 and 2 would be the main reviewers and theme generators. Reviewers 3 and 4 would act as a 'critical friend' later in the process to review and define themes.

Reviewer 5 would be brought in at the final stages to utilise his clinical expertise and ensure usability and translatability of our findings and recommendations towards clinical practice. After a further meeting with Reviewer 1 and 2, it was decided that the data analysis would certainly take time and be an inductive process. We were mindful from the start not to rush this process, and we continually re-visited the data. The data had thus far proved so contextually rich that we knew early that repeated re-reading, interpretation, and meetings would be required. Processes were subject to change as the data unravelled. Reviewer 1 kept an audit trail of processes and a list of common emerging themes from the outset so that we could review our timeline and present our methodology well. We decided to focus on First Visit Interviews to understand fully the nature of behaviours in fibromyalgia (FM) prior to delving into treatment responses.

The first five participants' interviews were to be utilised for initial theme generation. We analysed each interview separately in order to maintain uniqueness, before bringing it all together for theme generation. For each interview for each individual participant, a 3-column table was constructed. Reviewer 1 inputted all comments from Reviewer 1 and 2 into the first 2 columns and subsequently produced a combined summary of related comments. Reviewer 1 and 2 had a further meeting to agree upon initial themes and patterns that we had so far found from our initial dataset. At this point, we agreed on 5 overarching themes; 'biopsychosocial that influence thoughts and behaviour', 'interaction, identity, autonomy and support', 'journey, impact and change following FM onset', 'coping and management strategies', and 'views and perception of light therapy'. Themes were colour-coded in order to easily pick out and group comments for further analysis (Figure S2).

PARTICIPANT A FIRST VISIT		
**Reviewer 1 review**	**Reviewer 2 review**	**COMBINED SUMMARY**
Interaction between pain, confidence, memory combined with uncertainty of how fibro will be each day - which places most restriction on individual	Memory and confidence are the major issues participant associates with the fibro. Vicious cycle now developed as dependent on others due to low confidence.<br>Condition unpredictable. Allows pain to dictate the day<br>Lots of onus on whether people believe them, seeks validation	**Biopsychosocial that influence thoughts and behaviour:**<br>• ***Reduction in confidence associated with social interactions***: Confidence in social interaction and memory (forgetting what she is saying), worry around uncertainty of how bad fibro symptoms will be each day. Stigma influencing confidence of interactions<br>• ***Reduction in confidence associated with unpredictable nature of pain***<br>• ***Pain dictates the day***
How Fibro is seen impacts how they react to others or what they say to others – making support important as a factor that influences well-being<br><br>Rich detail of the impact and restriction placed – context between who they used to be active and now	Feels can't say when struggling as symptoms are there persistently and wasn't sure if just in head due to struggles with anxiety and depression<br><br>Strong sense of illness as identity, almost seems ashamed when talking about not leaving the house anymore<br><br>Longing for how used to be pre-diagnosis	**Interaction identity, autonomy and support**<br>• ***Decreased Confidence in revealing symptoms, due to stigma of condition and complexities like mental health problems***<br>• ***Past identity impacts willingness to talk about the negative thoughts and behaviour that resulted from having fibro***<br>• ***Restrictions placed by the condition and impact***<br>• ***Wanting restoration to pre-diagnosis***
Key evidence of a journey<br><br>Impact of surgery<br><br>Long time to diagnosis	Was not keen on looking for work as wanted to help daughter with baby. Now recognises work may increase her self-worth. Feels link between condition and redundancy → further loss of confidence. Blames unemployment on motivation levels and mental state, rather than pain<br><br>Puts diagnosis down to trauma of surgery ?physical or emotional. Recognises long delay in coming to diagnosis. Never heard of fibro prior to diagnosis – happy to have it validated.<br>Prefers not to socialise, makes excuses, defeatist attitude	**Journey, impact and change following fibro**<br>• Process of diagnosis<br>• Fibro created change and impact to job and activities and relationships<br>• Negative cycle created from loss of job on motivation, decisions and behaviour<br>• Reduction or change in social activities<br>• Understanding the impact and value of past interventions

Figure S2. Reviewer 1 and Reviewer 2 comments with patterns identified early.

Phase three: generating themes

Now that we had our overarching themes, we moved onto the next phase: developing a thematic table. From herein, participants beyond participants E would have their data inserted here, bypassing the 3-column table theme generation phase. The thematic table consisted of four columns. The first column was populated by the overarching initial themes generated. Column two was populated by Reviewer 1 during this next phase of data immersion. This was a relatively long process, which involved close analysis of all transcripts and resulted in a 69-page document. The third column incorporated the definition of the sub-theme, the uniqueness, the negative cases and quotes. The final column kept a record of participants who fit into this sub-theme during the interview. Each interview was analysed with a 'fine toothcomb' in turn, with continual population of this initial thematic table (Figure S3).

Theme	Sub-theme	Definition, Uniqueness, Example Quote	Participants
**Biopsychosocial that influence thoughts and behaviour**	**Pain and stiffness dictates activity** level (pain/tenderness)	Definition: this sub-theme represents unpredictable nature of pain controlling daily activities. The future can be seen as uncertain, because several aspects influence this including; (a) the experience of pain is unknown and uncertain (P01), the number of days affected each month varies (P02) (b) if pain levels are too high activity ceases in order to manage, because it hurts to move (P01;P15; P18; P21) and restricts activity (P02;P15; P16; P18; P21), (c) pain could affect certain parts of the body varying the impact it has (P01;P05;P10; P20), pain response disproportionate to activity levels (P13), use of NHS services during pain flares (P14), unpredictable impact on work duties (P18; P24), weather dictates pain and activity (P18)<br><br>Uniqueness:<br>Any groups more or less affected?<br>Negative cases:<br>Does anyone ever say they just embrace it, that it doesn't matter what it is like you have to carry on?<br><br>Quotes:<br>"I don't know how I'm gonna be tomorrow let alone anything else", "I could wake up and I'm that tired that I don't even wanna get dressed", "my pain's that bad…I just think – no, I can't be bothered", "it even hurts just to get upstairs", "pain varies at different times in different parts of my body", "I used to walk my labrador quite a distance across the canal – don't do it so much now because of the pain" [P01]<br><br>"I'm living with pain on a daily basis which is restricting me doing the things I've enjoyed", "some days are worse than others…in a month typically 2days, but could be 5/6 days it just varies" [P02]<br><br>"stiffness throughout the body, shaking in my hands, headaches which are quite severe, pain in my elbows, skin sometimes sore to the touch…sometimes I can't touch my head 'cause it's that sore. I get a lotta back pain, I can't sit down for long periods of time…my legs are really…it feels like pulsating pains down my legs in my muscles. Not been able to walk very well…tripping over things". "In a morning it takes me 3 hours just to get mobile 'cause everything's just stiff from the waist down…all these symptoms are every other day" [P03]<br><br>"very tight and aching as if somebody needs to roll my body out with a rolling pin. At first it's kinda like you've got the flu – I felt heavy and achey. Then it became more of a forward thought, like at first I could kind of push it to the back of my brain, then it became very much of a thought of 'OK that's hurting and I need to address that pain'", "random spasms, I don't know if it's because my body's so tense because of being so tired and in so much pain…it's as if it's twinging and spasming so I'll constantly have to move to get comfortable…sometimes when I don't get relief I'll just be pacing up and down. Or I'll try and crack my back and stretch it out but it just doesn't do anything", "there will be days where it just takes it toll and you can't, and then you feel	P01, P02, P03, P04, P05, P10, P13, P14, P16, P18, P20, P21, P22, P23, P24

Figure S3. Initial thematic table by Reviewer 1.

Phase four: reviewing potential themes

The initial generated themes were reviewed by the main reviewer. It was felt that too many themes had arisen secondary to the sheer abundance of data. These themes potentially overlapped, with some themes being over-descriptive while others were not obvious in meaning. The main themes were perhaps too prescriptive, with a subconscious attempt to slot datasets to fit with predefined OMERACT domains [55]. Sufficient time was allowed for processes and data analysis to be thoughtful and absorbing (>8 months). Some sub-themes were removed, as they were not found to have enough rich data patterns across participants, for example, 'attempting to seek out others', 'better suited to flexible employment'. Semi-objective measures were removed and reserved for prospective thematic analysis of treatment effects to enable comparisons to be clearly made.

Upon further review by Reviewer 2, themes and sub-themes were on the whole retained at this point, but with altered definitions of sub-themes and movement of quotes between sub-themes. On the odd occasion, the wording of the sub-theme was altered to be more descriptive, for example, 'mood and motivation dictate activities' was

changed to '*low* mood and motivation dictate activities'. Definitions at this point became more descriptive with examples. We recognise bringing in a second reviewer at this point is not strictly in keeping with reflexive thematic analysis. However, owing to Reviewer 1's background, we realised that the data would be looked at with a primarily scientific mindset. The benefit of bringing in Reviewer 2 brought in their strengths and vast experience of qualitative methodology and analysis.

It became evident that one sub-theme was leading into another, and we felt that we had gone beyond themes and could see processes developing during our data analysis phase. At this point, we did not yet know the precise process, and we were vigilant on developing these processes and recognising patterns in data. The patterns were not obvious and semantic; they were rather more latent, requiring us to delve deeper into understanding each participant. The sub-theme 'fatigue and sleep disturbance' was recognised as too broad and non-descriptive at this point. We recognised that confidence had already come up earlier and therefore should be grouped with confidence under the theme of 'identity, autonomy, interaction and support'. Again, the first two sub-themes here talk about confidence and could be merged.

Phase five: defining and naming themes

After agreeing on themes and sub-themes, the reviewers began to consolidate their thoughts on the processes seen and the interlinkage between the themes. Reviewers 1, 2 and 3 initially met to share ideas and agree on a model that illustrates the processes we were seeing across participants. It was agreed at this point that there was a definite negative spiral of events following FM diagnosis. Reviewer 2 proceeded to condense the wealth of data down into more manageable portions, with the quotes selected really highlighting the themes and sub-themes. A process map also begain to simultaneously emerge.

After multiple iterations of this initial model, Reviewers 2, 3 and 4 met, with Reviewer 4 acting as a 'critical friend' and developing the model further. It was here that the importance of multiple reviewers with diverse backgrounds really became apparent. Secondary to Reviewer 4's background in physiotherapy and related research, we drew upon this experience to introduce the World Health Organization's International Classification of Functioning, Disability, and Health (ICF) framework [16] as our 'Theme' descriptions. As we were adopting a pragmatist approach, and the main essential for us was to get across usability data to all healthcare professionals involved in treating patients with FM, using the ICF domains brought in the usability aspect to this analysis. Not least, because they break down FM nicely into easily targetable biopscyhosocial aspects of one's life. Something we all agreed upon at the outset was the need for this paper to really bring about some action points for clinicians going forward, or at least for researchers to target treatment ideas.

Phase six: producing the report

Following description of themes and sub-themes, it was clear that common processes were unfolding**.** Both Reviewers 1 and 3 derived at an early stage that FM seemingly sent the participant into a negative spiral. Participants perceive that healthcare professionals break FM down into manageable segments due to a lack of understanding, and for ease of treatment—it is easier to treat something in isolation than as a complex whole.
